# Hypertonic saline versus fluid restriction: The pitfalls in managing acute hyponatremia in a patient with long‐standing spinal cord injury

**DOI:** 10.1002/ccr3.6576

**Published:** 2022-12-02

**Authors:** Vaios Koutroukas, Panagiotis Pavlou, Emilia Imogen Smith, Andra Maria Ciutac, Christopher Redford, Jamie C. Smith

**Affiliations:** ^1^ Diabetes and Endocrinology Department Torbay and South Devon NHS Foundation Trust Torquay UK; ^2^ Faculty of Biology, Medicine and Health University of Manchester Manchester UK; ^3^ Present address: Royal Devon University Healthcare NHS Foundation Trust Exeter UK; ^4^ Present address: Wexham Park Hospital, Frimley Health NHS Foundation Trust Slough UK

**Keywords:** antidiuresis, fluid restriction, hyponatremia, spinal cord injury

## Abstract

Spinal cord injury‐induced hyponatremia is an under‐recognized entity, without a mention in the European hyponatremia guidelines. We present a case of a 56‐year‐old female quadraplegic patient with cervical cord injury, presenting with severe hyponatremia and tonic–clonic seizures. This case highlights the challenges in medical management; action mechanisms are further discussed.

## Abstract 2

In patients with spinal cord injury‐induced hyponatremia, it is important to maintain a careful fluid balance to allow adequate intake but avoid over‐hydration, backed up by judicious monitoring of electrolytes.

## 1 CASE HISTORY

A 56‐year‐old female quadriplegic patient presented to our emergency department with acute confusion followed by a tonic–clonic seizure. She had quadriplegia as a result of cervical (C_5/6_) cord injury sustained 28 years earlier, following a paragliding accident. There was no previous history of seizures. With regard to activities of daily life, she had a full package of daily care, mobilizing with the use of an electric wheelchair, with oral intake facilitated by spoons and straws. In terms of bladder and bowel hygiene, she had a long‐term suprapubic catheter, with a manual evacuation once every other day. Her physiological baseline included chronic hyponatremia averaging around 125 mmol/L, chronic anemia with a hemoglobin (Hb) at a level of 100 g/dl, a temperature of 34–35°C, a heart rate (HR) of 50–55 bpm, a systolic blood pressure (SBP) between 80 and 90 mmHg, and a diastolic blood pressure (DBP) between 30 and 40 mmHg.

Exploring the history leading up to her admission, she had felt generally unwell, reporting offensive urine for 3 days prior to her admission. She had received treatment with co‐amoxiclav and tigecycline for a suspected urinary tract infection (UTI) in the community. Her daily fluid intake was not considered to be excessive although she had been advised in the past to increase fluid intake when suspecting a possible UTI to mitigate the effects of bladder stasis.

## 2 INVESTIGATIONS

Initial blood tests on admission showed evidence of severe hyponatremia with a serum sodium concentration of 97 mmol/L; other laboratory investigations around the time of admission are shown in the Table 1.

**TABLE 1:** Admission blood investigations

Laboratory tests	Results	Reference ranges
Cortisol	1326 nmol/L	166–507 nmol/L
Sodium	97 mmol/L	133–146 mmol/L
Potassium	3.5 mmol/L	3.5–5.3 mmol/L
Urea	1.7 mmol/L	2.5–7.8 μmol/L
Creatinine	<18 μmol/L	45–84 μmol/L
Glomerular filtration rate	>90 ml/min	60–150 ml/min
Glucose (fasting)	4.7 mmol/L	3–5.4 mmol/L
Thyroid‐stimulating hormone	0.38 mIU/L	0.35–4.5 mIU/L
Total protein	54 g/L	60–80 g/L
Albumin	36 g/L	36 g/L
Total bilirubin	24 μmol/L	0–21 μmol/L
Alkaline phosphatase	47 IU/L	30–130 IU/L
Serum osmolality	225 mmol/kg	275–295 mmol/kg
Urine osmolality	406 mmol/kg	50–1200 mmol/kg
Urine sodium	52 mmol/L	–
Urine potassium	45 mmol/L	–
C‐reactive protein	51 mg/L	0–5 mg/L

## 3 INPATIENT MANAGEMENT

She was admitted to the intensive care unit (ICU) for management. Two intravenous 150 ml boluses of 2.7% saline were administered, followed by a daily fluid restriction of 750 ml. Changes in serum sodium over time in response to the above clinical management strategies are shown in the figure. Unfortunately, fluid restriction resulted in persistent hypotension (BP 50/25 mmHg) requiring vasopressor support with metaraminol. Subsequently, fluid restriction was relaxed to 1 L per day.

She spent 6 days in ICU before vasopressor support was successfully weaned down. She was subsequently monitored on a general medical ward for another 3 days and then discharged home with a serum sodium of 119 mmol/L and a fluid restriction of approximately 1 L daily.

## 4 OUTPATIENT MANAGEMENT

**FIGURE 1:**
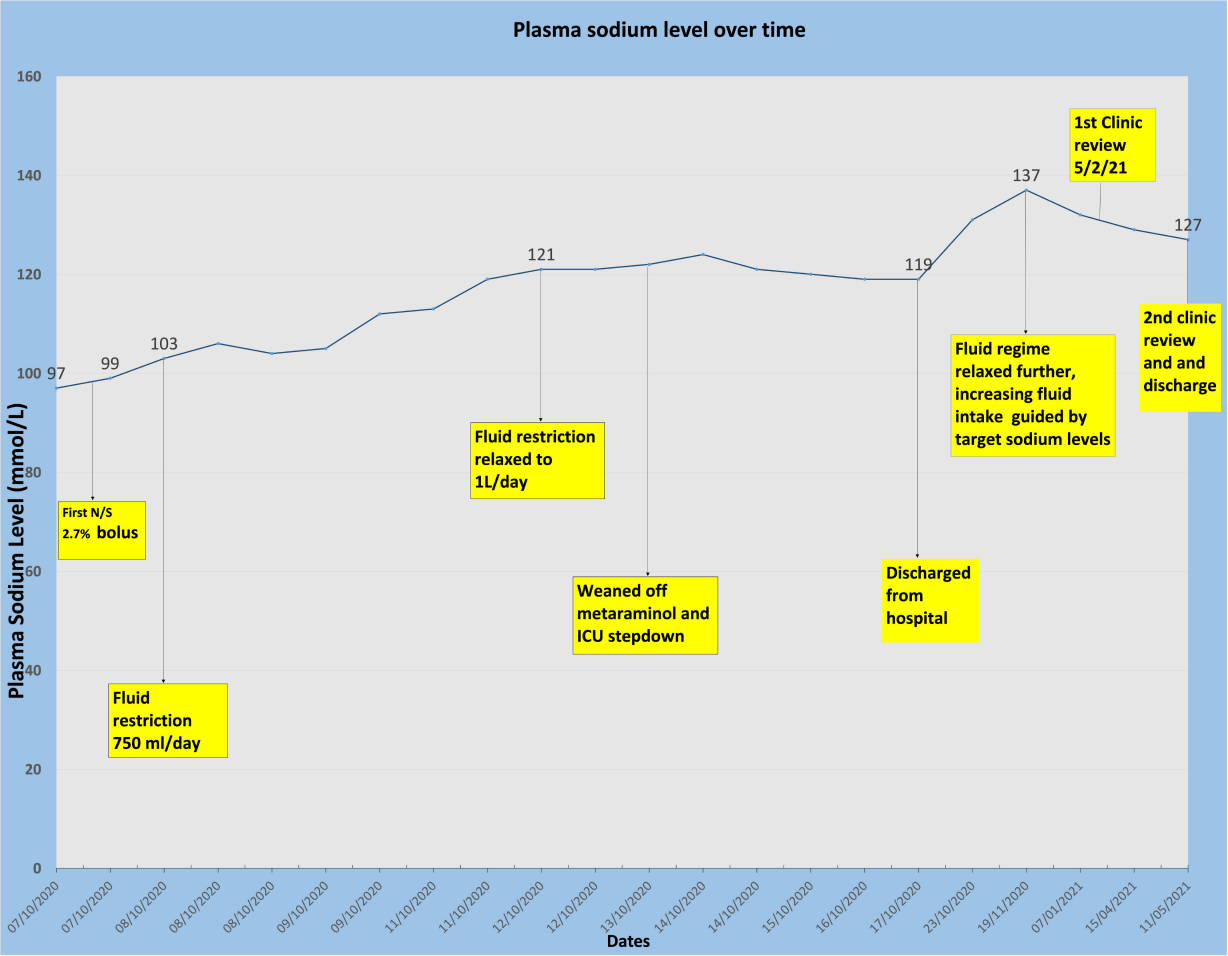
Plasma sodium level over time.

At outpatient clinic review 3 months later, blood tests showed serum sodium concentration had increased to 137 mmol/L with a relative rise in urea to 5 mmol/L. Although she had not become acutely unwell, the ongoing fluid restriction was presenting challenges, such as increased thirst and exacerbation of chronic hypotension, causing dizziness. There were also patient concerns over possible recurrence of urinary tract infections due to oliguria with bladder stasis. In light of what appeared to be an over‐correction in sodium concentration associated with reduced fluid intake and relative hypovolemia, she was advised to follow a more relaxed fluid regime with a target sodium level of 125–130 mmol/L. The patient's condition improved and serum sodium levels remained stable between 125 and 130 mmol/L with normal electrolytes and renal function and a reduced reported incidence of urinary symptoms (Figure 1).

## 5 DISCUSSION

As seen in the present case, the emergency use of hypertonic saline in acute hyponatremic encephalopathy is vital in order to reverse dangerous neurological complications due to cerebral oedema. Following careful raising of serum sodium, fluid restriction is an important therapeutic strategy used in euvolemic hypotonic hyponatremia to produce a further, slower rise in sodium.^1^, ^2^ This intervention was chosen in the present case but unfortunately led to unintended and deleterious consequences, in particular severe hypotension which required inotropic support. The underlying mechanisms which led to a worsening of our patient's clinical hemodynamic status might be explained by the nature of hyponatremia associated with SCI, a condition which has key pathophysiological differences to euvolemic hyponatremia due to SIADH.

Spinal cord injury (SCI) is a well‐established cause of clinically significant chronic hyponatraemia.^3^, ^4^, ^5^ Polyuria and compensatory polydipsia are partially explained by the effects of autonomic denervation on the kidneys' ability to conserve sodium.^6^ The pathophysiology is probably related to sympatholytic effects on renal tubular recovery of sodium, to the loss of blood pressure support through both the sympathetic and somatic nervous system lesions, and to stimulation of thirst by volume depletion, together with activation of the renin‐angiotensin system.^3^

In some ways, SCI‐induced hyponatremia, especially in relation to high cervical cord lesions, shares similarities with the cerebral salt wasting syndrome^3^, ^7^, ^8^; it can be regarded as a form of “myelopathic form” of salt wasting. In both scenarios, there is a reduction in extracellular volume and compensatory increase in vasopressin release which, although contributing to the occurrence of hyponatremia, could be regarded as “appropriate” as it represents a pathophysiological adaption designed to restore circulating volume. This is in contrast to SIADH when euvolemia is present and exaggerated vasopressin release is by definition inappropriate.^1^ Consequently, fluid restriction as a treatment modality often works well to generate a gradual rise in serum sodium in SIADH. In contrast, SCI‐induced hyponatremia is associated with relative hypotension and reduced extracellular volume^3^; therefore, medically induced fluid restriction deprives these patients of polydipsia, their principal defense against circulatory collapse.^3^, ^9^

In our view, the occurrence of SCI‐induced hyponatremia has received relatively less attention in the contemporary medical literature in comparison with cerebral salt wasting. This latter term by its definition tends to direct clinicians to intra‐cranial pathology rather than myelopathy. Furthermore, UK^2^ and European consensus guidelines^1^ on the management of hyponatremia do cover the management of CSW but do not allude to SCI, and this might tend to encourage clinicians to erroneously assume SCI‐induced hyponatremia is akin to euvolemic SIADH.

## 6 CONCLUSION

In summary, although SCI‐associated hyponatremia shares some biochemical characteristics with SIADH‐related hyponatremia, there are notable differences, namely the risk of hypotension and hypovolemia which can occur with the former. Consequently, the use of fluid restriction regimes which are regarded as first‐line in the management of hospitalized patients with euvolemic hyponatremia due to SIADH due to SIADH^1^, ^2^ should be considered with extreme caution in patients with SCI‐induced hyponatraemia.^3^, ^7^, ^9^ Careful fluid balance to allow adequate intake but avoiding over‐hydration backed up by judicious monitoring of electrolytes is key to avoid the risk of acute hyponatremic encephalopathy in such patients.

## AUTHOR CONTRIBUTIONS

Dr Koutroukas contributed to the literature search and the writing of the manuscript, edited the figure and the table as well as completed the submission process. Dr Pavlou undertook the writing of the manuscript as well as the designing of the figure and the table. Ms Smith and Ms Ciutac conducted the literature search, jointly with Dr Koutroukas. Dr Redford provided critical feedback on the manuscript, focusing on the clinical details of the case. Dr Smith supervised the work, edited the manuscript with useful suggestions about the phrasing and the structure, and provided critical feedback on the manuscript.

## FUNDING INFORMATION

None.

## CONFLICT OF INTEREST

The authors have no conflicts of interest to declare. All co‐authors have seen and agree with the contents of the manuscript, and there is no financial interest to report. We certify that the submission is original work and is not under review at any other publication.

## CONSENT

Written informed consent was obtained from the patient to publish this report in accordance with the journal's patient consent policy.

## Data Availability

No new data generated, or the article describes entirely theoretical research.
